# LATER-LIFE LIVING ARRANGEMENTS OF CHILDLESS AMERICANS: A LIFETABLE APPROACH

**DOI:** 10.1093/geroni/igz038.2328

**Published:** 2019-11-08

**Authors:** James Raymo, Xiao Xu, Jersey Liang, BoRin Kim, Mary Beth Ofstedal

**Affiliations:** 1 University of Wisconsin-Madison, Madison, Wisconsin, United States; 2 Yale University, New Haven, Connecticut, United States; 3 University of Michigan, Ann Arbor, Michigan, United States; 4 University of New Hampshire, Durham, New Hampshire, United States

## Abstract

The large body of research on living arrangements at older ages pays little attention to the growing population of childless men and women. We begin to fill this gap by using data from the Health and Retirement Study (HRS) over a period of 14 years (2000-2014) to describe the number of years between ages 65-90 that childless Americans live in four different living arrangements: alone, with spouse, with others, and in a nursing home. Because the number of childless HRS respondents is not large (n = 835 in 2000), we first estimate sex-specific single decrement life tables by race/ethnicity and by educational attainment. We then use Sullivan’s method to calculate living arrangement-specific life expectancy for each group, thus providing a comprehensive descriptive portrait of sociodemographic differences in living arrangements across older ages for childless Americans. Preliminary results show that differences in living arrangement-specific life expectancy by race/ethnicity and educational attainment primarily reflect group differences in mortality. The proportion of later life spent in different living arrangements is generally similar across racial/ethnic groups and education levels. This stands in contrast to large racial/ethnic and educational differences documented in earlier studies of older Americans with at least one living child. Results also show that the proportion (and years) of later life spent living alone is substantial, especially for women (over 50%). We discuss the potential implications of these findings with reference to both projected trends in the childless population and research on associations between living arrangements and health of childless older Americans.

